# Tribo-Electrochemical Mechanism of Material Removal Examined for Chemical Mechanical Planarization of Stainless-Steel Using Citrate Buffer as a Complexing Agent

**DOI:** 10.3390/ma18020317

**Published:** 2025-01-12

**Authors:** David R. Santefort, Kassapa U. Gamagedara, Dipankar Roy

**Affiliations:** Department of Physics, Clarkson University, Potsdam, NY 13699-5820, USA; santefdr@gmail.com (D.R.S.); gamageku@clarkson.edu (K.U.G.)

**Keywords:** chemical–mechanical planarization, tribo-corrosion, tribo-electrochemistry, stainless steel, removable surface film, salt film

## Abstract

Chemical mechanical planarization (CMP) is a technique used to efficiently prepare defect-free, flat surfaces of stainless steel (SS) foils and sheets that are implemented in various modern devices. CMP uses (electro)chemical reactions to structurally weaken the surface layers of a workpiece for easy removal by low-pressure mechanical abrasion. Using a model CMP system of 316/316L stainless steel (SS) in an acidic (pH = 3.63) slurry with alumina abrasives, citrate buffer (CB), and H_2_O_2_, we examine the tribo-electrochemical mechanisms of SS CMP that dictate the designs of functionally efficient and cost-effective CMP slurries. The use of CB as a pH-controlled complexing agent prevents defect-causing dissolution of SS and eliminates the need for using separate (often toxic) corrosion inhibitors in the slurry. A material removal rate of 8.6 nm min^−1^ is obtained at a moderate down pressure of 0.014 MPa with a platen rotation speed of 95 RPM. Electrochemical techniques are strategically combined with mechanical abrasion of SS test samples to probe complex CMP mechanisms that are not readily accessible with electrochemical experiments alone. Corrosion-like reactions of salt-film formation at the SS surface act to enable the CMP process, where corrosion-induced wear plays a major role in material removal.

## 1. Introduction

The use of stainless steel (SS) substrates has expanded over the last decade for applications in a broad range of devices, including solar cells [1], electronic displays [2], thin film transistors [3], and electrochemical sensors [4]. Adequate surface flatness is a critical requirement to maintain the functional qualities of these substrates, and chemical mechanical planarization (CMP) is a leading technique currently used to meet this requirement [5,6,7,8,9,10]. The general field of CMP has rapidly advanced in recent years following the enduring progress of CMP research in the field of microelectronics. While several industrial applications of SS CMP have already been identified [6,9], currently available reports of detailed research on this topic [5,6,9,11,12,13,14,15,16,17,18,19,20] are relatively limited compared to those dealing with metal CMP in the field of microelectronics. Thus, the fundamental knowledge necessary to advance the technology of SS CMP is at a relatively early stage of development at this time.

The basic strategy of CMP for processing SS substrates is essentially the same as that used for integrated circuit (IC) manufacturing. This strategy follows from Preston’s and Archard’s formulations of material removal rates (MRRs) due to material wear. In this description, $\mathrm{MRR}=K_{P}\mathrm{PV}$, where $K_{P}$ is the Preston coefficient, P is the polishing pressure applied to the workpiece, and *V* is the relative linear velocity between the workpiece and the pad used for polishing. The mechanical component of CMP is largely governed by the values of *P* and *V*, while the chemical role of CMP is embedded in the Preston coefficient, which has the form *K*_p_ = *K*_a_/*H*_s_, with *K*_a_ and *H*_s_ denoting, respectively, the dimensionless Archard wear coefficient and the sample’s mechanical hardness. The additives included in a CMP slurry are intended to reduce the value of *H*_s_ by (electro)chemically modifying the surface layers of the sample, so that these layers can be removed with moderate mechanical abrasion [21]. Repeated formation and removal of the surface materials enable the process of substrate planarization.

The requirements of SS CMP are somewhat less stringent compared to those used for metal/alloy components in IC fabrication. For instance, the down-pressure of SS CMP can often be raised (to 0.03–0.04 MPa) to boost MRRs [4] without risking damages to the substrate. Options for abrasive particles in SS CMP have a broader range than that commonly used for wafer CMP in IC manufacturing [12,16]. The polishing time for SS CMP can also be extended to ≥30 min for higher yield of material removal [12]. Nevertheless, restricting CMP-related surface defects such as scratches, residues, and localized erosions, while maintaining an adequate level of MRRs remains a main requirement of SS CMP, as it is in the case of wafer CMP.

The chemical component of SS CMP is functionally similar to that of metal CMP in IC fabrication. Specifically, surface modification (hardness-reduction) of a CMP sample is governed by corrosion-like electrochemical mixed potential reactions, where the mechanochemical effects of friction (tribo-corrosion) also play active roles in determining the MRRs [22,23,24]. However, the complexity of slurry formulations for SS CMP is rooted in the fact that a single slurry must address the broadly varying surface chemistries of multiple metals contained in the alloy. In other words, the surface modifier additives (oxidizing and complexing agents, in particular) in an efficient slurry formulation for SS CMP should chemically prepare all or most of the major constituent metals in the alloy for mechanically assisted material removal. Additionally, according to the general guidelines of slurry selection for defect-minimized CMP, this surface modification for material removal should be governed mostly by the abrasion of hardness-reduced surface layers rather than by uncontrolled (and potentially site-specific) dissolution of these layers [25,26].

Selecting slurry consumables to meet all the above criteria for SS CMP (in a cost-effective and environmentally suitable approach) is challenging, especially in view of the strong chemical resistance of SS. For instance, if a complexing agent selectively reacts only with certain metal sites of the alloy surface, material removal will be spatially inhomogeneous across the surface. Additionally, while using environmentally suitable complexing agents (like carboxylic and amino acids), low-pH slurries are generally preferred to those at high pH levels for supporting adequate material removal [27]. At the same time, essentially all metal components of SS tend to dissolve in this low-pH environment, yielding non-negligible etch rates compared to polish rates in CMP. To address these issues, and to develop the general strategy of slurry engineering for SS CMP in a quantitative approach, it is necessary to further advance the fundamental understanding of the material removal mechanisms that are linked to metal-specific and combined surface chemistries of CMP in the presence of surface abrasion.

The present work focuses on some of the aforesaid challenges and mechanistic aspects of CMP for 316/316L SS, a highly corrosion resistant alloy that has generated considerable interest recently due to its efficient function as a substrate for modern solar cells [28]. For a test slurry, we used H_2_O_2_ as an oxidizer with alumina abrasives, in combination with citrate buffer (CB) as a complexing agent and a pH adjuster to support an acidic environment (at pH = 3.63, determined by trials for optimized CMP). As demonstrated later in this report with experimental data, these slurry additives can largely meet the main requirements of SS CMP stated above.

Due to the electrochemical origin of material wear/removal, electroanalytical tools are ideal for probing the mechanistic aspects of SS CMP [12,22,29,30]. Some electrochemical investigations of SS CMP systems are currently found in the readily available literature [5,11,12,13]. These studies have generally employed the technique of potentiodynamic polarization (PDP), using stationary working electrode (WE) samples of SS in the absence of surface abrasion. In rare cases, these earlier experiments have included electrochemical impedance spectroscopy (EIS) and open circuit potential (OCP) monitoring in a stationary state [31]. The technique of electrochemical mechanical planarization (e-CMP) has also been used to planarize SS substrates with voltage activated surface modifications [32]. Totolin et al. have studied tribo-electrochemistry of CMP for AISI 304 SS employing dynamic contacts of ball-on-disk and pin-polishing-cloth combinations [10].

In our present study, electrochemical measurements are combined with mechanical abrasion to mimic the tribo-electrochemical conditions of an actual CMP system. This is accomplished by integrating a commercial polisher into a custom-built test cell equipped with independent mechanical controls (*P* and *V*) of the sample interface, while the latter is monitored with electrochemical probes [26]. These electrochemical techniques include PDP, EIS, linear polarization resistance (LPR), and intermittent OCP transients.

Intermittent OCP transients (a widely used technique in tribo-electrochemistry [33]), PDP, and LPR measurements are performed both in the absence and in the presence of surface abrasion. Comparisons of the results obtained from these experiments with and without surface abrasion help to examine how the mechanical and chemical components of CMP support the process of material removal. EIS is used to examine the mechanism of surface film formation in the absence of active abrasion with and without the usual down-pressure of polishing being applied at the pad–sample contact. Thus, the present experiments are designed to probe the detailed mechanisms of material removal in a way that is not possible while using conventional electrochemical measurements without involving CMP specific elements of tribology. In experimental studies of SS CMP, these tribo-electroanalytical methodologies have mostly remained unexplored with respect to those generally found in the current literature on these CMP systems [5,6,12,13,14,16,17,18,19,27].

## 2. Materials and Methods

### 2.1. CMP Sample and Polishing Slurries

Type 316/316L stainless steel (SS316/316L) (McMaster-Carr, Elmhurst, IL, USA) disks of 2.54 cm diameter mounted in a water-tight Teflon holder were used as CMP samples, which also served as the WEs for electrochemical measurements. The elemental compositions of the SS samples are listed in Table 1 using median values of the alloy components. Each SS sample was polished using 600 grit sandpaper, working through 1000, 1500, 2000, 2500, 3000, and 5000 grit sandpapers in that order until a mirror finish was achieved, and then polished with a dampened polishing pad with 1 μm alumina powder (both from Buhler, Lake Bluff, IL, USA). SS316 is known to develop a passive layer of Cr_2_O_3_ in storage [17]. The initial polishing step removed this passivation layer; the sample was then thoroughly rinsed to remove any remaining alumina and dried before being placed in the test cell.

The slurries were at pH 3.63 and utilized 0.1 M KNO_3_ (Fisher Scientific, Waltham, MA, USA) as a background electrolyte, 0.1 M citrate buffer (CB) [based on citric acid (C_6_H_8_O_7_), Fisher Scientific, Pittsburg, PA, USA, and sodium citrate, Sigma-Aldrich (Darmstadt, Germany)] as a complexing agent, 1 wt% H_2_O_2_ (Fisher Scientific, Waltham, MA, USA) as an oxidizer, and 3 wt% 0.3 μm alumina (Buehler, Lake Bluff, IL, USA) as an abrasive. Each slurry formulation contained a reference (Ref) base of 0.1 M KNO_3_ and 0.1 M CB. As noted in the introduction, the implementation of CB in this study was not only intended as a pH buffer, but also as a complexing agent to control dissolution. The latter strategy was based on the observation that citric acid formed metal complexes with the main components, Fe [19] and Cr [20], of the SS alloy.

The CMP enabling components of the present slurries meet the general criteria of environmental compatibility. They do not include corrosion inhibitors many of which are linked to various toxic effects [34]. As a pH adjuster, CB is environmentally preferred to inorganic acids like HNO_3_ [35]. In its dual function as a complexing agent and pH adjuster, CB eliminates the need for using separate complexing agents such as nicotinic acid or ethylenediamine, and inorganic pH adjusters like H_2_SO_4_ or ammonium hydroxide [12,16,31]. CB, as a complexing agent, falls in the general category of “green” chemicals currently considered for SS CMP [12,13,18,20,27]. A further useful function of citrate ions released by CB is to keep alumina abrasives effectively dispersed in the slurries [36]. The oxidizer H_2_O_2_ can be considered as a green reagent [37]. Alumina powders (used here at an average dispersion of 0.3 μm) represent a recyclable class of abrasives frequently used for SS CMP [17,18,19]. Based on these considerations, the overall features of environmental compatibility for the present test slurries are comparable to those previously reported for SS CMP using carboxylic or amino acids as complexing agents, in combination with H_2_O_2_ as an oxidizer and alumina [17,18] or silica [12] abrasives.

A summary of the test slurries and their chemical combinations is provided in Table 2. Since these CMP slurries also served as electrolytes for tribo-electrochemical experiments, it was necessary to determine the slurry (solution) resistances (*R*_s_) for the analyses of electrochemical data. *R*_s_ was measured using EIS, with the SS sample held ~1 mm distance above the polishing pad in each experimental slurry. These measured values of *R*_s_ are included in Table 2.

### 2.2. Instruments and Measurements

The design and operation of the tribo-electrochemical test cell have been described elsewhere in full detail [26]. Briefly, a Struers Labopol Benchtop polisher (Struers LLC, Cleveland, OH, USA) was coupled with a slurry reservoir that served as the main cell chamber. The configuration of the samples within each slurry consisted of four different variations, as described in Table 3. We will use these designations to refer to the different mechanical arrangements of the test samples considered here. The polisher was controlled using LabVIEW software (version 16.0f5-2016, National Instruments, Austin, TX, USA) codes developed by our group. The polisher platen was covered with an IC1000 pad (Rohm and Haas, Newark, NJ, USA).

The sample-head and the platen were rotated at a common speed of 0 RPM (hold) or 95 RPM (polish/rotate), with a down-pressure of 0.014 MPa (2 psi) applied at the sample-pad interface. In situ electrochemical experiments were performed using a three-electrode setup where the sample was the WE and a stainless-steel ring surrounding the interior wall of the platen submerged in the slurry was the counter electrode (CE). A saturated calomel electrode (SCE) was the reference electrode (RE), connected via a salt bridge. All electrochemical measurements were performed using a Solartron 1287 potentiostat and its accompanying Frequency Response Analyzer 1252A (Ametek Scientific Instruments, Berwyn, IL, USA).

Etch (dissolution) rates for the abrasive-free slurries were tested in magnetically stirred slurries using a cleaned SS sample and gravimetric measurements. Material removal rates (MRR) were determined for all slurries with gravimetric measurements [38]. The instrumental limit on the accuracy of mass differences measured to determine the etch rates and MRRs was 0.01 mg. Standard errors in the measurements of removal rates based on the instruments and the procedures used here typically varied from ~10% of the mean MRR when the latter was recorded above ~3 nm min^−1^ to ~30% of the mean MRRs registered below ~3 nm min^−1^ [39]. The MRR values are reported here in the format broadly used in experimental studies of SS CMP [6,9,14,17,18,19].

Two types of linear sweep voltammetry (LSV) experiments were performed based on voltage sweep ranges: (i) Within the range of thermal voltage, centering around the equilibrium open circuit potential (OCP), in the mode of linear polarization resistance (LPR) measurements; (ii) extending the voltage range in the Tafel region for potentiodynamic polarization (PDP) measurements. OCP and LSV (both LPR and PDP) data were obtained for each sample configuration, along with EIS for the Up-Hold and Down-Hold cases. Intermittent (Down-Polish vs. Down-Hold) OCP transients were recorded with the sample for all slurry types. For slurries that also contained abrasives (slurry II and IV), additional OCP transients were obtained for the sample in the Up-Rotate and Up-Hold configurations. All PDP data were collected with a 5 mV s^−1^ scan rate within a potential range (typically ±0.5 V) centered about the sample’s OCP.

EIS data were obtained with the SS samples at their OCPs in the Up-Hold and Down-Hold cases, using a 10 mV (root mean square) sinusoidal perturbation within a logarithmically spaced frequency range of 1 Hz to 10 kHz. Repeatability of these measurements (reflecting the electrochemical stability of the tested systems) was experimentally verified by collecting three repeated data scans the results of which are presented in Figure S1 included in the Supplementary Material. Statistical uncertainties in the data were evaluated in the form of percentile standard errors in each of the impedance elements extracted from the EIS data (as further elaborated in Section 2.3).

### 2.3. Data Analysis Protocols

All PDP data were corrected to account for the effects of slurry (solution) resistances *R*_s_ that existed between the WE and the RE of the test cell. This was accomplished by using EIS-measured values of *R*_s_ in the formula $E_{e}=E-IR_{s}$, with $E_{e\;}$, *E* and *I* denoting the corrected and measured potentials, and the measured electrode current, respectively. Since $E_{e}=E$ for *I* = 0, the OCP (*E*_OC_) data were taken directly from the measured potentials. The slurry resistances used for ohmic corrections of PDP data were for Up-Hold sample configuration. This resistance changed in the sample-Down mode due to the addition of an interfacial resistance of the pad–sample contact. In the analyses of tribo-electrochemical results, the slurry resistances (*R*_sc_) measured in the pad–sample contact mode were treated as a variable of the polisher setup, as discussed elsewhere [40].

Origin software (version 2024) was used for Tafel extrapolations of *IR*_s_-corrected PDP data, as well as for other parts of data analyses and plotting of the figures. EIS data, processed as Nyquist impedance plots, were subjected to complex nonlinear least square (CNLS) fitting using ZSimpWin software (version 3.50). Electrically equivalent circuit (EEC) models of the CMP interface under different measurement conditions were developed from these analyses. The statistical error in each of the impedance elements obtained from these EECs was also determined through CNLS calculations in the approach previously discussed by Boukamp [41]. Detailed values of these errors (restricted at <10% values in most cases) are presented in Table S1 in the Supplementary Material.

## 3. Results and Discussion

### 3.1. Results of Material Removal Rate Measurements

**Figure 1:**
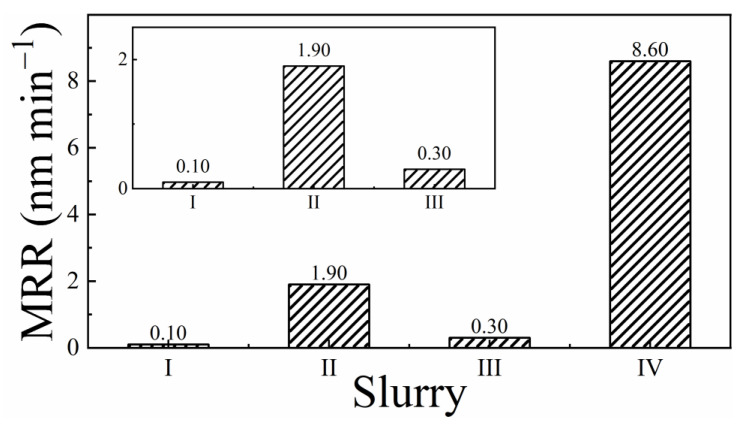
Material removal rates of a type 316/316L stainless-steel sample in slurries I–IV (slurry compositions are listed in Table 2). The number in each case denotes the corresponding numerical value of MRR (in nm min^−1^). The inset shows the low-MRR cases for slurries I–III on an expanded scale. The highest MRR is observed for slurry IV, which has all the components necessary to effectively form and remove CMP enabling surface films.

MRRs of the SS sample measured in the different test slurries are shown in Figure 1. The inset shows the MRRs for slurries I, II, and III, where the values are on a lower scale compared to slurry IV. Both slurries that contain alumina (II and IV) have MRRs roughly 20 times those of their respective slurries without alumina (I and III). The addition of H_2_O_2_ to the system supports a larger removal rate in slurry III compared to slurry I, with the MRR greatly enhanced due to the inclusion of alumina in slurry IV promoting abrasion-assisted material removal. No measurable etch rates (ERs) were detected in the dissolution tests. This indicated that material removal in all these slurries occurred mostly through the mechanical abrasion of insoluble species, rather than by the chemical dissolution of the CMP surface. This mode of material removal with suppressed dissolution is generally preferred to promote the planarization efficiency of CMP [25,42].

The down pressure used to obtain the MRRs in Figure 1 was set near the lower end of polish pressures commonly employed in the CMP of SS. This low-pressure setting was used to adequately bring out the chemical role of CMP and to facilitate the previously known conditions for minimizing CMP-generated surface scratches [43,44]. Experimental samples commonly used to report MRRs and etch rates of SS in the CMP literature (as in this work) are based on commercially available SS alloys that are fabricated for a broad range of applications. Quantitative comparisons of absolute removal rates for such samples are difficult considering the possible variations in material properties (specifically hardness) associated with the different manufacturing processes used. Nevertheless, for a comparative assessment of the present slurries’ overall function in material removal, it is useful to examine how the observed MRRs scale with respect to reductions in the polishing pressure and velocity. While the specific wear rate (SWR) of CMP is a suitable mechanical variable for this comparison, calculation of SWR requires certain information about the polisher’s dimension (in addition to the platen/sample angular velocity, *V*_Ω_) [39]. Since this dimensional detail of CMP polishers is not commonly reported with MRR values, the product, *PV*_Ω_, is often used as a process parameter to normalize MRR values with respect to the pressure and velocity of polishing [6]; here, we use this latter approach.

Jiang et al. have reported MRRs of 40–100 nm min^−1^ for SS CMP performed with *P* = 0.042 MPa (6 psi) and *V*_Ω_ = 150 RPM [12]. Similarly, using *P* = 0.02 MPa (3 psi) and *V*_Ω_ = 80 RPM, Hu et al. have reported an MRR of 42 nm min^−1^ for SS 304 in a slurry at pH = 2.6 with H_2_O_2_ and silica [6]. In our present study, *P* = 0.014 MPa (2 psi) and *V*_Ω_ = 95 RPM, which yield a maximum MRR of 8.6 nm min^−1^. By scaling this result with respect to the aforesaid values of *P* and *V*_Ω_ used by Jiang et al., a projected MRR of >40 nm min^−1^ is expected for slurry composition IV. Additional considerations for sample-specific grain-size differences [45] and compositional variations suggest that the MRRs obtained in this work are in the typical range for common applications of SS CMP. The data in Figure 1 also show how the MRRs can be varied by varying the oxidizer and abrasive (as well as complexing agent) concentrations in the slurry. The remainder of this report focuses on exploring the underlying mechanisms of these slurry-dependent MRRs.

### 3.2. Modes of Material Removal

To examine the tribo-electrochemical basis of SS CMP, it is useful to set up a phenomenological framework for data analysis based on the different modes of material removal. The following expression is frequently used to account for the different components of corrosion and wear in the CMP of alloys and metals [46,47]:

(1) $$MRR=[R_{c}+\;R_{wc}]\;+R_{cw}+R_{w}$$

where $R_{c}$ and $R_{w}$ denote the rates of material removal due to (electro)chemical corrosion and mechanical wear, respectively. $R_{wc}\;$ and $R_{cw}$ are material removal rates supported by wear-induced corrosion and corrosion-induced wear, respectively. The first two terms within square brackets on the right-hand side of Equation (1) correspond to the CMP mode of corrosion-like surface modification, which involves the formation of passive surface films as removable materials, sometimes in combination with direct dissolution (static and/or dynamic etch) [48,49]. $R_{w}$ corresponds to material removal from the chemically unaffected regions of the CMP surface, and, based on hardness considerations, the contribution of this term to the measured *MRR* remains rather small in most cases of metal/alloy CMP [50].

In the presence of dissolution, the effective chemical component of *MRR* can be described in the form:

(2) $$R_{c}(P)=ER\;(P)\;+{x[\;r}_{f}(P)]$$

where ER (P) is the etch rate and $r_{f}(P)$ is the corresponding rate of insoluble surface film formation under surface polishing. x is a scaling factor (formation: removal); if $R_{f}$ is the rate of surface film removal by abrasion, then $x=R_{f}/r_{f}$(P). When tribo-electrochemical processes are activated by surface abrasion, the underlying corrosion-like reactions of ER and ${\;r}_{f}$ are affected by mechanical wear and by dynamic stress of local variations in *P* and *V* [22,51]. Furthermore, the values of both ER and $r_{f}$ can increase if the thermal energy of friction due to surface abrasion acts to lower the activation energies of the surface reactions linked to these rate-terms [52,53]. At the same time, the down-force of polishing can lead to intergranular stress corrosion (an effect broadly known in the context of steel corrosion [54,55]), which then affects the values of both ER  and ${\;r}_{f}$.

The combined mechanical effects of polishing on ER(P) and ${\;r}_{f}(P)$ can be lumped into the term $R_{wc}$ of Equation (1). In view of the above considerations, the component (${MRR}_{corr}$) of material removal controlled by corrosion-like surface modification has the form: ${MRR}_{corr}\approx$ $R_{c}$+ $R_{wc}\;\equiv \;ER(P)\;$ + ${x[\;r}_{f}\left(P\right)]$. Thus, Equation (1) can be expressed as:

(3) $$\;\;\;MRR=ER(P)\;+{x[\;r}_{f}\left(P\right)]\;+R_{cw}+R_{w}$$

which explicitly includes the role of CMP-enabling surface films in determining the *MRR*s.

The contribution of $R_{cw}$ to the *MRR* can be significant, depending on the CMP system. The effects associated with this term often operate in sample regions adjacent to (but not within) the electrochemically modified surface layers, and, hence, remain undetected in electrochemical measurements of corrosion variables [38]. The mechanisms of these effects can include elastic mismatches between the modified and unmodified surface materials [56,57,58] and accelerated propagation of corrosion fatigues and dislocations [59], as well as corrosion-induced reduction in the local threshold for plastic deformation [60] in regions adjacent to chemically modified layers.

In the description of Equation (3), the data in Figure 1 demonstrate the expected result that x increases as abrasives are included in the slurry. Since the value of *x* in Equation (3) can be time-dependent during polishing, it is difficult to separately measure ${MRR}_{corr}$. However, this component of material removal is linked to the electrochemically measurable surface corrosion rate, *CR(P)*, of the CMP sample:

(4) $$CR\left(P\right)≅\;ER(P)\;+r_{f}\left(P\right)$$

under polishing. A similar expression for *CR(H)* follows from Equation (4), with ER(P)  and $r_{f}\left(P\right)$ replaced by ER(H)  and $r_{f}\left(H\right),$ respectively. If the CMP process operates in a steady state with the rates of surface film formation and generation being mutually balanced (*x* = 1), then ${MRR}_{corr}≅CR\left(P\right)$. The extent of mechanically affected (wear-induced) corrosion for a given CMP system can be examined in terms of the tribo-corrosion rate (*TCR*), defined as [38]:

(5) $$TCR=CR\;\left(P\right)-CR\;\left(H\right)$$

and by measuring the difference, [$MRR-CR\left(P\right)]$, it is possible to obtain an overall estimate of the material removal contribution from $R_{cw}.$

### 3.3. Surface Reactions of CMP

The surface reactions responsible for ${MRR}_{corr}$ govern the (electro)chemical efficiency of alloy/metal CMP, and thus constitute an essential aspect of crafting and evaluating slurry formulations for these systems. Usually, the reactants and products of these corrosion-like reactions remain unchanged between the H and P situations, while the reaction rates tend to change (generally by increasing) in the polish stage as the coverages of site-blocking surface species are reduced, and, in some cases, the frictional energy of abrasion alters the activation energies of CMP reactions [26]. To examine these reactions for the systems studied here, we concentrate on the elements representing >1% composition of the SS sample used, namely, Fe, Cr, Ni, and Mo (Table 1). According to published Pourbaix diagrams, all these metals in the acidic slurry environment at pH = 3.63 tend to dissolve as their respective cations [61,62,63,64]. 

For Fe [65,66], Cr [63], Ni [67], and Mo [64], the dissolution reactions are as follows:

Fe = Fe^2+^ + 2e^−^ (6)

Cr = Cr^3+^ + 3e^−^ (7)

Ni = Ni^2+^ + 2e^−^ (8)

Mo = Mo^3+^ + 3e^−^ (9)

and in an acidic CMP slurry, these anodic steps are driven by cathodic reduction of oxidizers in the mixed potential mode. In the absence of H_2_O_2_, this cathodic step in CMP slurries is generally dominated by the oxygen reduction reaction (ORR) [49],

O_2_ + 4H^+^ + 4e^−^ = 2H_2_O (10)

while in H_2_O_2_-containing slurries, the predominant cathodic reduction step at the CMP interface is [68]:

H_2_O_2_ + 2H^+^ + 2e^−^ = 2H_2_O (11)

which is then accompanied by an auxiliary cathodic step of ORR. The mixed potential forms of Equations (6)–(9) with Equation (10) are:

(12) $$\mathrm{Fe}+2H^{+}+\frac{1}{2}O_{2}={\mathrm{Fe}}^{2+}+H_{2}O$$

(13) $$2\mathrm{Cr}+6H^{+}+\frac{3}{2}O_{2}={\mathrm{Cr}}^{3+}+3H_{2}O$$

(14) $$\mathrm{Ni}+2H^{+}+\frac{1}{2}O_{2}={\mathrm{Ni}}^{2+}+H_{2}O$$

(15) $$2\mathrm{Mo}+6H^{+}+\frac{3}{2}O_{2}=2{\mathrm{Mo}}^{3+}+3H_{2}O$$

which operate in the H_2_O_2_-free slurries I and II used here. Likewise, the individual mixed forms of reactions (6)–(9) with reaction (11) are:

(16) Fe + 2H^+^ + H_2_O_2_ = Fe^2+^ + 2H_2_O

(17) 2Cr + 6H^+^ + 3H_2_O_2_ = 2Cr^3+^ + 6H_2_O

(18) Ni + 2H^+^ + H_2_O_2_ = Ni^2+^ + 2H_2_O

(19) 2Mo + 6H^+^ + 3H_2_O_2_ = 2Mo^3+^ + 6H_2_O

and these latter reactions play leading roles in material removal from the SS sample in the H_2_O_2_ containing slurries III and IV. Reactions (12)–(15) are expected to serve as secondary contributors to the material removal process in the latter slurries.

Although reactions (12)–(19) correspond to the dissolution of SS, the lack of measurable ERs suggests that the metal cations produced by these reactions undergo further steps to form undissolved complexes at the SS sample’s surface. Such two-step reactions involving intermediate species of dissolved metal cations are known to generate surface complexes in the form of solid salt films [69,70,71]. In acidic solutions, these salt films represent a commonly observed corrosion feature of electrochemically active SS surfaces [72,73,74,75,76].

When a metal/alloy surface dissolves in an electrolyte environment, metal cations released from the surface gradually diffuse out of the electrochemical double layer into the bulk solution [71]. Solid salt films are deposited on such a dissolving surface if the dissolved cations leave the double-layer region at a rate of diffusion that is considerably slower than the rate of dissolution [77]. In this situation, the sample–solution interface becomes supersaturated with the dissolved species. If salt-forming anions are present in the interfacial solution, metal cations react with these anions to form salt films, which subsequently deposit onto the metal/alloy substrate.

Given that the slurries are at pH 3.63, the complexing agent species that is most predominant is H_2_Cit^−^, mixed with a much lower amount of H_3_Cit, where Cit ≡ C_6_H_5_O_7_ [40]. Reactions of H_2_Cit^−^ with the aforesaid metal cations can deposit a mixed layer of hydrated metal salts containing FeHCit [78], CrCit [79], NiHCit [80], and MoH_3_Cit_2_ at the SS surface. The reactions of salt film formation in this case are:

Fe^2+^ + H_2_Cit^−^ = FeHCit + H^+^ (20)

Cr^3+^ + H_2_Cit^−^ = CrCit + 2H^+^ (21)

Ni^2+^ + H_2_Cit^−^ = NiHCit + H^+^ (22)

Mo^3+^ + 2H_2_Cit^−^ = MoH_3_Cit_2_ + H^+^ (23)

and the composite film on the SS surface would contain a mixture of these citrate salts. Based on the SS sample’s elemental composition (Table 1) and relative values of the natural release rates of its constituent metals [81], the citrate salts of Fe, Ni, and Cr are expected to dominate the makeup of the surface film. If the interface remains supersaturated with the metal cations, the salt film’s solid phase should be largely retained [71]. In the CMP situation, the composite salt film of SS would then serve as a removable material by friction. Additionally, surface layers underlying the salt film would be structurally weakened due to localized defect pits caused by the film’s formation [70,73,75], and would also serve as a removable material by abrasion [69].

The foregoing discussion of CMP reactions provides a mechanistic framework to explain the MRRs observed in the different slurries. In the H_2_O_2_ free slurries I and II, reactions (16)–(19) are absent. The amount of salt film formed only by the ORR-supported reactions (12)–(15) in this case is not sufficient to sustain an adequate rate of material removal. Furthermore, polishing the SS sample without abrasive particles in slurry I does not provide the frictional force necessary to fully abrade the affected surface layers of SS. As a result, the MRR in slurry I remains at a lower value than that recorded in the H_2_O_2_-free slurry II with abrasives. In the H_2_O_2_-based slurries III and IV, both sets of reactions, (12)–(15) and (20)–(23), simultaneously operate to generate adequate levels of wear to support material removal. Consequently, the MRRs measured in slurries III and IV yield larger values than those of their counterparts in slurries I and II. Once again, the abrasive-containing slurry IV supports a higher MRR than that of the abrasive-free slurry III (Figure 1). This occurs due to inefficient friction of surface polishing in the latter case.

### 3.4. CMP Reactions Probed with Electrochemical Impedance Spectroscopy

A major mechanistic factor of SS CMP in this study is the slurry-specific electrochemical nature of salt films formed at the CMP interface. EIS is an established tool to confirm the formation and to study the detailed features of salt films at dissolution-prone electrochemical interfaces [82,83,84]. EIS was performed to examine the SS interface in the different slurries under OCP conditions, with the sample placed in both the Up-Hold and Down-Hold arrangements. Results of these two sample configurations were compared to test how the polishing pad’s pressure at the sample surface affected the films formed. The stationary sample-pad setup was used for these measurements, since stable EIS data for all four slurries could not be obtained in the presence of surface abrasion. The collected data were useful for comparison with previously published EIS results, most of which employed stationary samples and unstirred bulk solutions (in three-electrode arrangements electrochemically comparable to the Up-Hold configuration used here).

**Figure 2:**
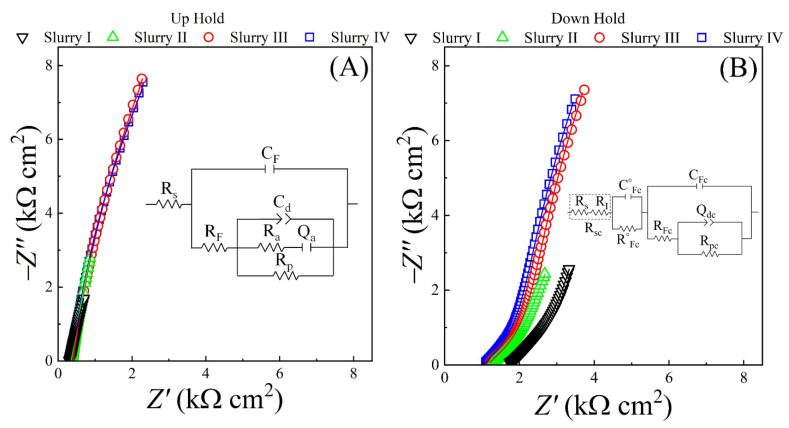
Nyquist impedance plots for the SS sample collected using the Up-Hold (**A**) and Down-Hold (**B**) configurations in each slurry listed in Table 2. The sample configurations are defined in Table 3. The geometric sample area of the SS sample was used to normalize both the real (Z′) and the imaginary (−Z′′) components of the system’s complex impedance, *Z*. The insets show the EEC models of the CMP interface obtained from CNLS analyses of the EIS data. The symbols and the lines denote experimental data and CNLS fits to the data, respectively.

Figure 2 shows EIS-recorded Nyquist impedance plots for the SS sample in the different slurries used. The symbols in Figure 2 are data points and the lines denote CNLS fits with the resulting EECs for the Up-Hold and Down-Hold cases shown in Figure 2A and Figure 2B, respectively. Table 4 presents the impedance variables (discussed below) obtained by CNLS fitting the EIS data, along with certain related parameters derived from the EEC elements. The plots for the different slurries in the Up-Hold case in Figure 2A display mutually very similar features, and, hence, largely overlap on each other. However, the “lengths” of the individual plots in Figure 2A are different for the different slurries, which indicates that (the impedance contributions and) the timescales of the reaction steps associated with these plots are slurry-specific. For the Down-Hold case, the Nyquist plots in Figure 2B exhibit relatively more prominent differentiable features among the four slurries.

Both the EECs shown in Figure 2 contain the known signature features of salt films associated with dissolving solid surfaces [85,86]. The overall composition of the EEC in Figure 2A is commonly found for metal and alloy electrodes coated with porous, passive films, and this model also applies to salt films formed at electrode surfaces in stationary bulk electrolytes [85,87]. The EEC is commonly associated with surface films that have relatively uniform composition and density across their thickness.

In the EECs shown, *R*_s_ is the solution resistance, while *C*_F_ and *R*_F_ denote, respectively, the capacitance and resistance formed by pores in the salt film. *R*_p_ is a polarization resistance, which, in the present study, arises from the mixed potential reactions of salt film formation. *R*_a_ and *Q*_a_ denote nonfaradaic adsorption of anions at the SS surface [39], where *Q*_a_ is characterized by two CPE parameters, *Y*_a_ (amplitude) and *a* (index). The impedance elements in Figure 2B are labeled with a subscript “c” corresponding to the sample-pad contact mode, where the SS surface is pressed against the polishing pad. In the latter situation, the double-layer capacitance takes the form of a CPE, *Q*_dc_, the impedance of which is composed of two CPE parameters, *Y*_dc_ and *d*_c_.

In the contact mode, the slurry solution stored in the pad’s pores and grooves serves as the electrolyte [88,89]. The polishing pad used in this work contain pores with diameters varying in the 30–70 μm range, and occupy ~30% of the pad’s volume [90]. The element *R*_I_ represents the ohmic resistance of the interfacial slurry that resides within the pad’s porous volume. *R*_I_ adds to *R*_s_ and forms a net series resistance *R*_sc_, denoting the effective solution resistance in the contact mode (*R*_sc_ = *R*_s_ + *R*_I_) [40]. It is useful to note here that in the zero-frequency limit, the total impedance of the EECs in Figure 2A,B has the following finite values: (*R*_s_ *+ R*_F_ *+ R*_p_) and (*R*_sc_ *+ R°*_Fc_ *+ R*_Fc_ + *R*_pc_), respectively. In the infinite-frequency limit, these EEC impedances also maintain finite values, *R*_s_ and *R*_sc_ in Figure 2A and Figure 2B, respectively. Thus, both EECs meet the EIS validation criterion of finite impedance at extreme frequency limits [91].

The two series-connected main blocks of impedance elements after the solution resistance elements in Figure 2B are typical of salt films that have packing densities (and sometimes compositions) varying in a direction normal to the dissolving surface [82,86]. In such cases, the impedance block next to the solution resistance can be associated with a porous film of low density, while the next group of elements can be taken as a model of a compact, less permeable film. The porous film supports usual ionic transport in the solution phase. The right-most block of impedance elements in Figure 2B is essentially the same as the main EEC block in Figure 2A, except that the adsorption branch is absent in the Down-Hold case. This larger block likely represents the compact region of the salt film that is adjacent to the SS surface [82,92]. The [*C°*_Fc_ – *R°*_Fc_] combination can be associated with a loosely formed region of the salt film on the slurry side of the electrochemical interface [71,82]; *C°*_Fc_ and *R°*_Fc_ are the capacitance and the ohmic resistance of this relatively “open” film, respectively. In certain cases where the compact portion of the surface film becomes largely nonporous, the mechanisms of ion transfer in the two series-connected film regions tend to be different [92]. In these cases, a high-field mechanism [69,93] governs ion conduction in the compact film, while the porous region maintains the usual modes of diffusion and migration [89].

In the present case, the compact film below the porous region also remains measurably porous, as indicated by the finite-valued pore resistance, *R*_FC_, in the EEC component of this film. Therefore, ion transport throughout the overall salt film’s thickness should be dictated by liquid-phase ion transport via migration and diffusion. Ateya and Pickering have theoretically treated this mechanism of ion transport and salt film formation at stationary dissolving surfaces [94]. According to their model, the ratio of diffusion and migration fluxes in a salt film during its formation plays active roles in determining the film’s structural details. Additionally, the ion concentration at the outer plane of the electrode’s diffusion layer affects the film’s impedance characteristics. These detailed features of diffusion and migration change as the SS sample’s placement changes from the Up-Hold to the Down-Hold configurations (with the pad asperities being pressed down onto the SS sample in the latter case). Owing to these effects, and as suggested by the obtained EEC models, the surface film transitions from a single-phase composition in the Up-Hold case to a dual-phase structure in the Down-Hold situation.

**Figure 3:**
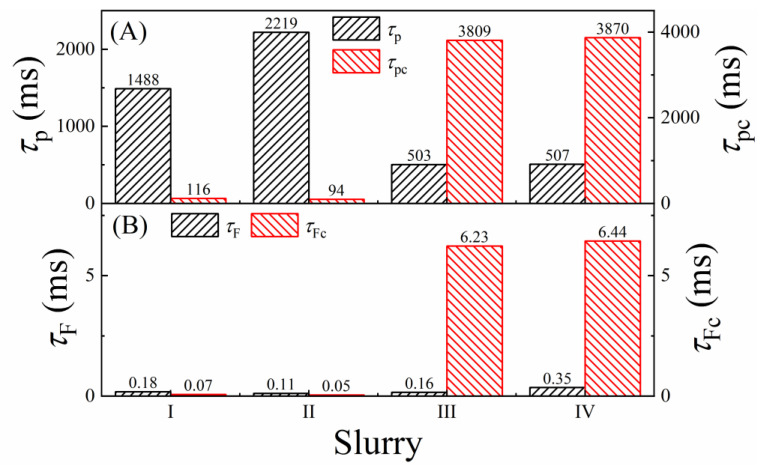
Characteristic relaxation times for (**A**) mixed potential reactions leading to removable surface (salt) film formation and (**B**) surface film stabilization. The left and right vertical panels in each panel represent results for the Up-Hold and Down-Hold sample configurations, respectively. The number associated with each bar represents the value denoted by that bar in its corresponding unit shown on the vertical axes. Slurries numbered I-IV on the X-axis have been defined in Table 2, and the plots here are based on the data shown in Table 4 for these slurries.

Further mechanistic details of the CMP enabling surface films of SS can be found in the slurry-dependent trends of the impedance variables. While the full set of these variables are included in Table 4 for completeness, the derived EIS parameters of primary interest in the present context are the time constants, *τ*_F_ (*τ*_Fc_) and *τ*_p_ (*τ*_pc_), plotted in Figure 3. Here, *τ*_F_ and *τ*_Fc_ are the relaxation times of the CMP-enabling surface films in the Up-Hold and Down-Hold configurations, respectively. The values of *τ*_F_ (*τ*_Fc_) can be approximated as the timescales for reactant transport through the salt film’s porous structure. The values of *τ*_p_ (*τ*_pc_) provide a measure of the time constants for mixed potential reactions being affected by charge relaxation in the double layer.

The variables measured in the sample-pad contact mode (Down-Hold) are labeled with a subscript “c”. The time constants are calculated using the variables in Table 4 as follows: *τ*_F_ = *C*_F_*R*_F_; *τ*_p_ = *C*_d_*R*_p_; *τ*_Fc_ = *C*_Fc_*R*_Fc_; and *τ*_pc_ = *C*_dc_*R*_pc_. Here, *C*_dc_ is an equivalent capacitance of *Q*_dc_, estimated as follows [95]:

(24) $$C_{dc}={\left(Y_{dc}{R_{pc}}^{1-d_{c}}\right)}^{\frac{1}{d_{c}}}$$

with the parameters from Table 4. The effects of slurry chemistries on these EIS-measured time constants are examined in Figure 3.

When present in the alumina-based slurries, H_2_O_2_ increases the concentration of hydroxyl groups Al-OH and Al-O-OH on the abrasive particles [96]. In bulk slurries, the heavily hydroxylated particles tend to block the salt film’s porous channels, which is manifested as increased values of *τ*_F_ found in slurry IV compared to those measured in the other slurries (Figure 3B). The plotted values of *τ*_Fc_ show that this combined effect of alumina and H_2_O_2_ does not cease to operate in the SS sample’s contact mode, where material transport occurs in a restricted space [97]. In the presence of H_2_O_2_, the surface films formed in the Down-Hold are substantially thicker than those formed in the Up-Hold case. This is seen in the increased values of *R*_Fc_ for slurries III and IV compared to those of slurries I and II (Table 4). This thickening of the surface film under constrained transport of reactants (specifically metal cations) is drastically reflected in the substantially affected values of *τ*_Fc_, which make *τ*_Fc_ (slurries III, IV) >> *τ*_Fc_ (slurries I, II).

The $R_{p}$ ($R_{pc}$) values dictate those of $\tau_{p}{(\tau }_{pc}).$ The slurry-dependent variations of *τ*_p_ are nearly reversed in the case of *τ*_pc_. In alignment with the results for *τ*_F_ and *τ*_Fc_, the comparative values of *τ*_p_ and *τ*_pc_ suggest that the salt films deposited at the stationary SS interface have different structures between the Up-Hold and Down-Hold situations. These differences can be attributed mainly to the different amounts of diffusion spaces available to the salt-forming species in the two cases [97]. Specifically, in the Down-Hold case, owing to the larger interfacial concentrations of dissolved metal cations in the constrained interfacial space, the surface films can be deposited in densely packed structures. This effect is further boosted in the H_2_O_2_-containing slurries III and IV, where the metal dissolution reactions are enhanced [94]. This effect is manifested in the slurry-dependent values of *τ*_pc_, which are consistent with the observed trends of *τ*_Fc_.

The time constant *τ°*_Fc_ linked to the relatively less compact salt film (developed between the bulk slurry and the compact surface) was calculated as: *τ°*_Fc_ = *C°*_Fc_*R°*_Fc_. As this film is formed, the timescale for its stabilization is registered in the charge–discharge time constant, *C°*_Fc_*R°*_Fc_. In the H_2_O_2_-free slurries I and II, the concentration of dissolved metal cations is limited. Consequently, the loosely packed outer region of the salt film takes relatively longer time to stabilize. In the H_2_O_2_-containing slurries III and IV, the diffusion and migration of dissolved cations increase, which in turn allow for relatively faster formation and stabilization of the (outer region of the) film. These effects can be noted by comparing the values of *τ°*_Fc_ for slurries III and IV with those of slurries I and II.

The intercepts of the Nyquist plots on the Z′ axis in Figure 2A,B represent the effective slurry resistances *R*_s_ and *R*_sc_, respectively. As seen in Table 4, the *R*_s_ values are mostly comparable among the four slurries. The rightward shift in the Down-Hold Nyquist plot between slurries II and I (Figure 2B) can be linked to a change of *R*_I_ as the alumina powder is introduced in the interfacial slurry. This change can be seen from the difference in the effective solution resistances between the Down-Hold samples in slurries that did not contain alumina (slurries I and III) and the slurries that did (slurries II and IV).

Salt films formed at dissolving alloy surfaces in aqueous media introduce superficial defect sites in their underlying layers; this causes spatial inhomogeneities at the interface, which are manifested here in the forms of the CPEs *Q*_a_ and *Q*_d_ detected in the Up-Hold and Down-Hold cases. While the film itself is a readily abradable material [for the term *r*_f_ in Equation (1)], its underlying defect sites can act as active spots to initiate mechanically propagated wear under continued surface abrasion in CMP [60]. Depending on the extent of this wear propagation, the latter material can have strong contributions to the MRR of CMP via the term *R*_cw_ in Equation (3).

### 3.5. CMP Mechanism Examined with Linear Polarization Resistance Measurements

**Figure 4:**
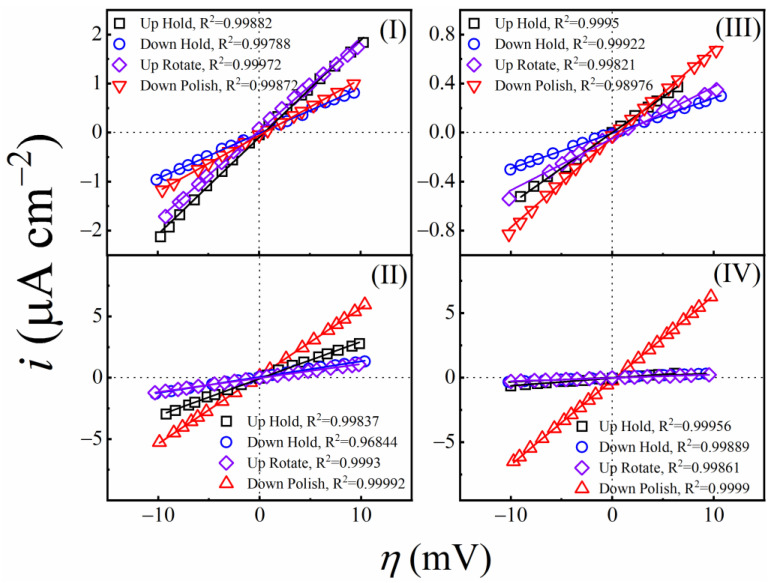
Linear polarization plots of type 316/316L stainless steel in slurries (**I**–**IV**), shown in the correspondingly labeled panels. The overpotential (*η*) range is ±10 mV in each case. The data points are shown as symbols while the lines represent linear fits with each coefficient of determination (*R*^2^ value) listed alongside their respective graphs.

While the foregoing EIS results center mostly on the formation characteristics of CMP supporting surface films, comparative tribo-electrochemical measurements, with and without surface abrasion, are necessary to investigate the removal aspects of these films. LPR measurements were performed for this purpose, and the results are shown in Figure 4. These experiments used LSV to record the current density (*i*) vs. overpotential (*η = E* − *E*_OC_) profile of the CMP interface within a small range of *η*, both in the presence and absence of surface abrasion. The symbols and the lines in Figure 4 denote experimental data and linear fits to the data, respectively. The four plots in each panel correspond to the four sample configurations described in Table 3. The range (±10 mV) of *η* used here is less than the operative thermal voltages (~26–27 mV, depending on the hold or polish situations), and, hence, the applied voltage serves mostly as a probe of the CMP interface rather than affecting the CMP process.

**Figure 5:**
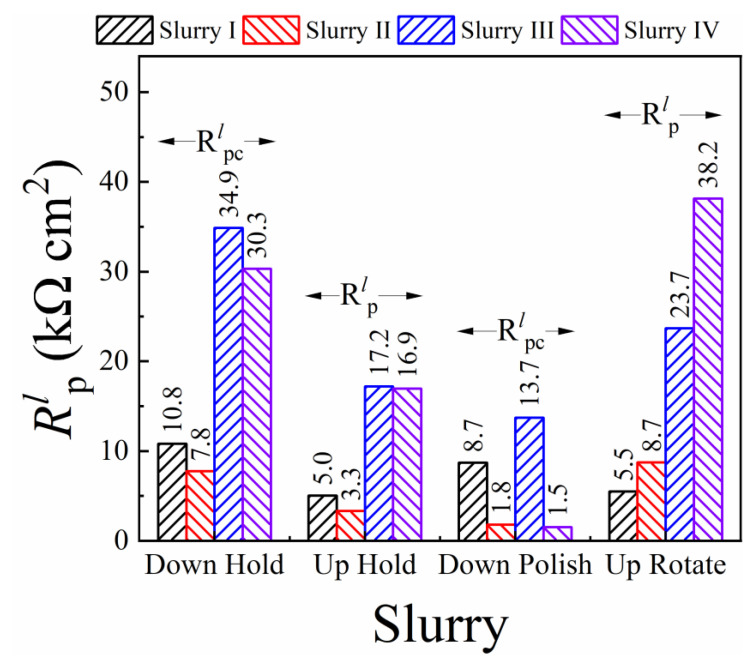
LPR values (*R^l^*_p_ and *R^l^*_pc_, respectively, for sample-up and sample-down arrangements) determined using the slopes of the linear plots in Figure 4 for slurries I–IV. Down conditions were enabled by applying a downward pressure of 0.014 MPa (2 psi) on the SS sample surface and Up conditions were applied by lifting the SS sample off the polishing pad by 1 mm. The comparative values of *R^l^*_p_ and *R^l^*_pc_ are used as indicators of SS surface passivation due to salt film formation.

The slopes of the linear plots in Figure 4 are used to determine the effective linear polarization resistance (LPR),

(25) $$R_{p}^{l}={\left(\frac{\partial i}{\partial \eta }\right)}^{-1}$$

where the value of *R^l^*_p_ is dominated by that of *R*_p_. This LPR also contains contributions of anion adsorption [98] as well as those of the other voltage dependent elements connected in parallel with *R*_p_. Thus, *R^l^*_p_ is a measure of the overall faradaic response of the CMP interface; a separate notation is used here for this resistance to mark its difference from *R*_p_ measured with EIS under stationary conditions at the OCP. Under surface abrasion, the LPR is denoted here as *R^l^*_pc_. The comparative values of *R^l^*_p_ and *R^l^*_pc_ provide a basis for evaluating the effectiveness of abrasion in material removal. These comparisons are considered in Figure 5.

As seen in Figure 5, LPR (Down-Hold) > LPR (Up-Hold) for all four slurries, and this shows how the polishing pad’s presence at the sample surface limits the effective area where the sample-slurry electrochemical interactions operate. For the abrasive based slurries, it is also found that LPR (Down-Hold) is slightly lower than LPR (Up-Rotate), that is the electrochemically passivating surface films are somewhat more compact and/or thicker for the Up-Rotate system. This shows that the hydrodynamic conditions supported at the sample surface by rotating the sample-platen combination in a non-contact mode have no detectable effects of flow-accelerated corrosion or film-thinning (an effect observed for certain systems involving particle-laden fluid flow) [99,100]. The electrochemically passivating surface film in the abrasive based slurries grows in a fairly effective way under the dynamic conditions where convective transfer of citrate ions from the solution to the sample surface is activated.

In the transition from Down-Hold to Down-Polish, the LPR values exhibit notable drops in all the experimental slurries. This provides evidence that the surface passivating salt film, which is responsible for producing high values of LPRs, and serves as a removable material for CMP, is effectively removed by abrasion. An observation of specific relevance in this context is that the polish-supported drop in the value of *R^l^*_pc_ is most pronounced in the case of slurry IV, where H_2_O_2_ is present to favor the formation of salt films, and alumina abrasives are also present to augment the friction of polishing. This finding reinforces the CMP mechanism proposed here, implying an active role of citrate ions in the formation of surface films at the SS surface. This finding also confirms the essential role of alumina abrasives in material removal, as noted in the proposed CMP mechanism.

### 3.6. Probing Tribo-Electrochemistry of CMP Using Intermittent OCP Transient Measurements

**Figure 6:**
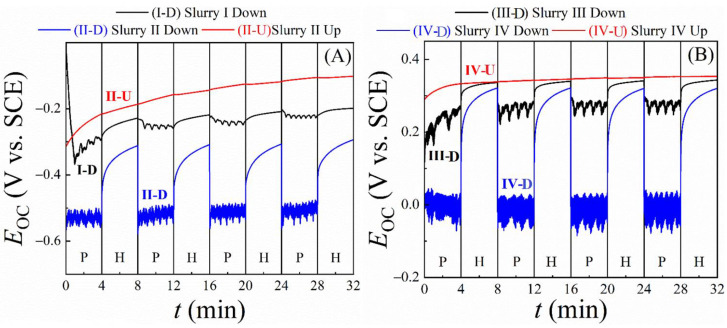
Open circuit potential transients compared for slurries I and II (**A**) and slurries III and IV (**B**). Each sample was switched between a polish (P) and hold (H) state in 4 min intervals for a total of 32 min (4 cycles). The slurries without abrasives (I and III, black) were only tested with the sample pressed into the polishing pad. The slurries that contained abrasives (II and IV) were tested with the sample pressed onto the polishing pad (blue) and lifted off the polishing pad without contact (red).

The OCP of a CMP system represents the equilibrium mixed potential of the interface where currents generated by electron transfer from the WE to a solution species (cathodic currents) are balanced by the currents generated by electron transfer in the opposite direction (anodic current). Variations in the OCP indicate how the surface processes affect this equilibrium potential according to the operative CMP mechanisms. Thus, in the general field of tribo-electrochemistry [101], as well as in studies of CMP mechanisms [26,102,103], it is a common practice to measure OCP transients by altering the system through the application and withdrawal of a mechanical perturbation at the surface being tested. Following this scheme, OCP transients involving Down-Hold vs. Down-Polish conditions were measured in the present investigation. The same measurements were carried out for the Up-Rotate sample configuration using slurries II and IV to check the possibility/extent of the slurry-fluid’s impact with abrasives included in the fluid [28]. The results of these intermittent OCP (*E*_OC_) transients measured in the H_2_O_2_-free and H_2_O_2_-containing slurries are shown in Figure 6A and Figure 6B, respectively.

The sample-up OCP data are minimally affected by the sample’s rotation. The slowly rising OCP profiles recorded in these cases represent correspondingly increasing thicknesses of surface passivating salt films that gradually approach saturation values. Absence of flow-induced corrosion in the present system has already been implied by the LPR data in Figure 5. This observation is further supported by the OCP data in Figure 6 since the sample-up OCP profiles did not register any distinctly separable features in response to alternating between stationary and rotating sample conditions. The sample-up OCP data in Figure 6 also help to identify the source of the rapid potential fluctuations (noise) that are superimposed on the Down-Polish OCP profiles. Since no interfacial currents operate at the OCP, any roles of fluctuating solution resistances (via the ohmic drop *IR*_s_) in generating the OCP noises during sample rotation can be ruled out.

Another possible source of voltage noise can be the rotating sample (even without an electrode current) if the rotation causes fluctuations in equipotential surfaces connecting the reference and working electrodes. This effect should mostly remain unchanged between the Up-Rotate and Down-Polish situations, since the rotational configuration and the sample-pad vertical alignment remain the same between these two cases. However, the OCP noise is only seen for the Down-Polish setting in Figure 6, while the corresponding plots of the sample-up OCPs are essentially noise-free with and without rotation. Therefore, the OCP noises are strictly polish-induced, and are unrelated to changes in equipotential surfaces and/or other possible sources of instrumental artifacts. Based on earlier reported results for similar experimental systems [22,33,56], the OCP fluctuations observed in Figure 6 can be attributed to the noise of tribo-corrosion (tribo-noise) caused by rapid passivation and activation of electrochemically functional surface sites of the SS sample [56]. This attribution is consistent with and provides further support for the CMP mechanism considered here in the framework tribo-corrosion based on surface reactions.

For a majority of metal and alloy surfaces controlled under tribo-corrosion, it is commonly found that the higher the value of the OCP, the lower is the faradaic activity of the associated interface [104,105]. According to this provenance, the OCP data-trends seen in Figure 6 are fully aligned with the CMP mechanisms discussed here in the context of Equations (12)–(23) to explain the slurry dependent MRR data in Figure 1. For instance, in all cases of Figure 6 where the sample is pressed against the pad, there is a jump between the hold (H) and polish (P) states. The OCP is consistently higher in the H state compared to that in the P state and, based on the aforesaid association of OCP values with surface passivation, these data indicate the presence of relatively thicker surface films in the hold case. When the sample enters a polish state with abrasives, a quasi-steady condition is established where surface passivation due to salt film formation is largely balanced by abrasion (removal) of the film.

The range of OCP variations measured between the H and P states increases when going from slurries I and II to slurries III and IV. The addition of H_2_O_2_ in slurry III provides a higher efficiency of salt film formation than CB by itself in slurry I. Consequently, the removal of these films causes a relatively larger drop in the OCP values in these slurries. When the sample enters a hold state, the passivating salt film grows at the SS surface. Without abrasives, the difference between P and H states is still observed, but to a much lesser extent, and in the case of slurry I, an upwards trend is initially seen as time progresses. The smaller change between states for these samples indicates that the surface film formed under stationary hold is not removed to the same extent as those with the abrasive present. This demonstrates once again that the abrasive particles are essential for efficient mechanical removal of the modified surface layers of SS, as H_2_O_2_ is essential to support the (electro) chemical process of this surface modification.

### 3.7. Results of Tribo-Potentiodynamic Polarization Measurements

**Figure 7:**
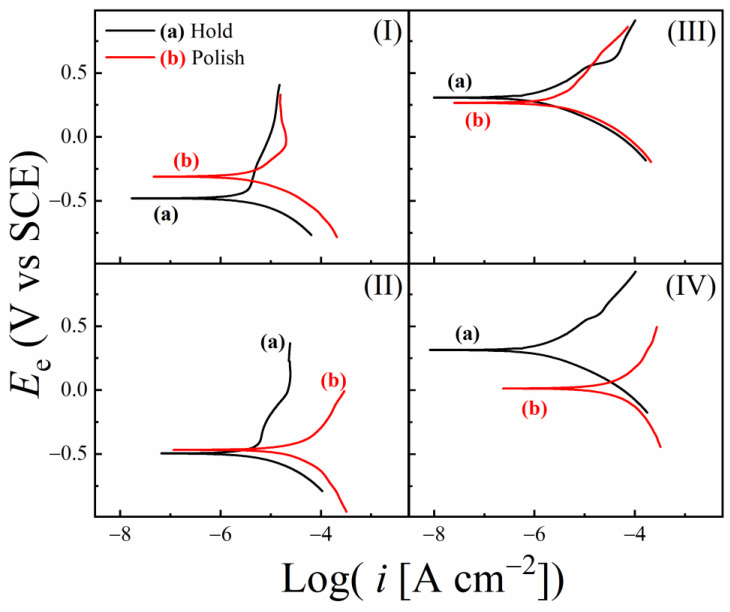
Potentiodynamic polarization plots for type 316/316L stainless steel in slurries (**I**–**IV**), shown in the correspondingly labeled panels. The vertical axis represents *IR*_s_-corrected potentials, *E*_e_. Plots (a) and (b) in each panel correspond to Down-Hold (H) and Down Polish (P) sample configurations, respectively. The relative placements of the upper and lower current branches of each PDP plot on the current axis represent, respectively, the overall strengths of the SS sample’s anodic and cathodic activities.

Tafel plots, recorded using PDP experiments and corrected for solution resistances for slurries (I)–(IV) are shown in Figure 7(I)–(IV). In each panel, the data for the Down-Hold (H) and Down-Polish (P) sample configurations are shown as plots (a) and (b), respectively. Ohmic corrections for slurry resistance were applied to the electrode potentials by using EIS-measured values of *R*_s_ from Table 4. The primary goal of conducting these PDP experiments was to measure the slurry-dependent rates of corrosion and tribo-corrosion, and to examine, using the framework of Equation (3), if and how these rates were correlated with their corresponding MRRs.

None of the polarization plots in Figure 7 contains any clearly identifiable features of strong surface passivation. Thus, it is evident from these data that the surface films supported on the SS sample under the given experimental conditions are porous, and that faradaic steps can be sustained in the presence of these films by interfacial charge transfer through the films’ porous channels. The polarization plots at hold shows no major dependance to the presence of abrasive particles [comparing plots (a) for slurries I vs. II and III vs. IV]. Any slight variations found in these comparisons could be linked to small changes in the effective surface pressure on the SS sample due to the absence or presence of alumina particles at the pad–sample interface.

**Figure 8:**
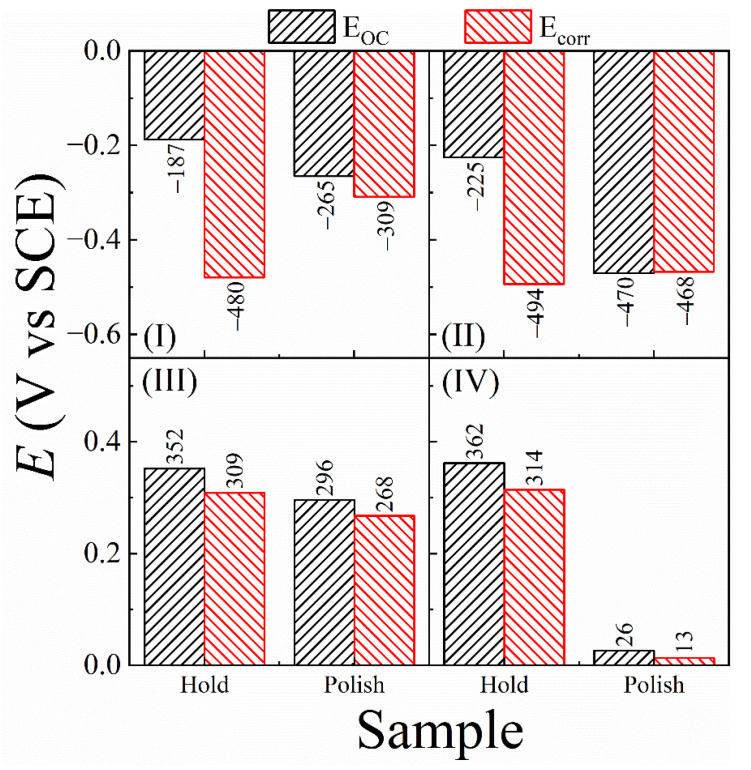
*E*_OC_ and *E*_corr_ values compared for each sample and slurry combination. The *E*_OC_ values were recorded following the procedures used to collect data for Figure 6. The *E*_corr_ values were obtained through extrapolation of the sample’s respective Tafel plots in Figure 7. The number associated with each bar denotes the corresponding plotted value in V vs. SCE. The differences between the *E*_OC_ and *E*_corr_ values in each case represent a measure of voltage activated surface adsorption by reaction intermediates in PDP. Panels (I), (II), (III) and (IV) represent data for the correspondingly numbered four slurries defined in Table 2.

The corrosion potentials (*E*_corr_) and corrosion current densities (*i*_corr_) were obtained from Tafel extrapolations of the PDP data. Corrosion rates (CRs) and tribo-corrosion rates (TCRs) of the SS sample in the different test slurries were determined from the corrosion currents. The *E*_corr_ measured using PDP essentially represents the same equilibrium potential as that measured in the form of *E*_OC_(for Figure 6) without applying polarization. However, the values of *E*_corr_ and *E*_OC_ for CMP systems tend to differ from each other due to their different measurement conditions. Due to this reason, and for the purpose of consistently analyzing the data using different measurement techniques, it is necessary to check how the values of *E*_OC_ and *E*_corr_ compare for each of the test slurries. This comparison is considered in Figure 8, where *E*_OC_ values (taken from Figure 6) for the different slurries are compared with their corresponding *E*_corr_ data (compiled from Figure 7).

In Figure 8, the polish vs. hold trends of *E*_OC_ for the H_2_O_2_ containing slurries III and IV are largely maintained for those of *E*_corr_, whereas the corresponding trends are not maintained between the *E*_OC_ and *E*_corr_ data collected in the H_2_O_2_ free slurries I and II. Under voltage activation in acidic media, Fe as well as Ni sites of the SS surface sites faradaically react with adsorbed water molecules to form insoluble oxide surface species of these metals [106,107]. It is now evident that, in the absence of H_2_O_2_, the reactions of salt-film formation are inefficient; if the SS electrode is polarized in this situation, the reactions of oxide formation would dominate the faradaic response of the SS surface. Specifically, as the electrode potential is anodically scanned, faradaic oxidation of Fe starts at −0.1 V with the step: Fe + xH_2_O = 2x (H^+^ + e^−^) + FeO_x_, where x has a typical value of 1.1 [108]. Within the voltage scan range considered in Figure 7, this FeO_x_ further oxidizes, where the typical reaction product has the makeup of Fe_2_O_3_.

Thus, the SS surface during its LSV scan in slurries I and II develops Fe_2_O_3_ species that are mixed within the salt film. When this surface is polished without abrasives during the LSV scan in slurry I, some of the structurally weakened surface material is removed as evidenced in the slight rightward shift in the anodic Tafel branch of (b) with respect to that of (a) in Figure 7(I). If the anodically generated Fe_2_O_3_ is not removed by this relatively weak abrasive-free polishing, the residual Fe_2_O_3_ on the SS surface acts as a catalyst for the ORR [109]. As expected, based on this scenario, under combined actions of polishing a voltage scan, an increased cathodic activity of the SS surface can be seen in the higher cathodic currents of plot (b) compared to those of plot (a) in Figure 7(I).

In the H_2_O_2_-free slurry II, the surface species of Fe_2_O_3_ forms again on the SS surface. The alumina abrasives included in this slurry facilitate effective removal of the salt film as well as some of the anodically produced Fe_2_O_3_. Like the salt film, Fe_2_O_3_ appears to acts as an anodic suppressor [110]. Thus, combined removals of the salt film and the Fe_2_O_3_ species under abrasive-supported polishing increase the currents on the anodic Tafel branch from plot (a) to plot (b) in Figure 7(II). The cathodic branch of plot (b) in this case also contains somewhat higher currents than those of the corresponding plot (a); this latter effect can be attributed to the extra cathodic component of ORR promoted by the catalytic effect of the residual Fe_2_O_3_ left on the SS surface. The cathodic and anodic shifts of *E*_corr_ caused by these combined effects are largely balanced, which results in the nearly equal values of *E*_corr_ for plots (a) and (b) in Figure 7(II) and Figure 8(II).

As seen in Figure 8(III,IV), the *E*_corr_ data recorded in slurries III and IV exhibit essentially the same (Down-Polish vs. Down-Hold) trends of their corresponding *E*_OC_ data. This indicates that the H_2_O_2_-activated reactions (16)–(19) in these cases dominate the surface chemistry of SS even when LSV is activated. The anodic production of Fe_3_O_4_ appears to be a slow process that remains suppressed by comparatively faster reactions of H_2_O_2_. As a result, the predominant surface species formed on the SS surface in the PDP experiments involving H_2_O_2_ [Figure 7(III) and Figure 7(IV)] are the same as those discussed for these slurries in the context of the OCP data in Figure 6B; the only difference between the OCP and PDP experiments is that, in the latter case, the anodic and cathodic components of the mixed potential reactions occur under controlled voltage activation.

The mutually comparable polarization plots (a) and (b) in Figure 7(III) indicate that polishing without alumina abrasives in slurry III is ineffective in removing the PDP-generated surface complex layers. In slurry IV, H_2_O_2_ favors the formation of anodically blocking salt films, and the pad’s abrasion with alumina efficiently removes this film (and its affected regions). Thus, theTafel plot (b) in Figure 7(IV) for the SS surface under polishing appears to show considerably higher anodic currents compared to those of plot (a) collected under stationary hold. At the same time, the cathodic branches of plots (a) and (b) remain mostly comparable, which is expected as the production of cathodically active (ORR-catalyst) Fe_2_O_3_ remains suppressed in both cases by the competing H_2_O_2_-augmented reactions of salt film formation. Thus, the resulting net shift in *E*_corr_ from (a) to (b) agrees with the corresponding results for *E*_OC_.

### 3.8. Comparison of the Rates of Corrosion, Tribo-Corrosion, and Material Removal

**Figure 9:**
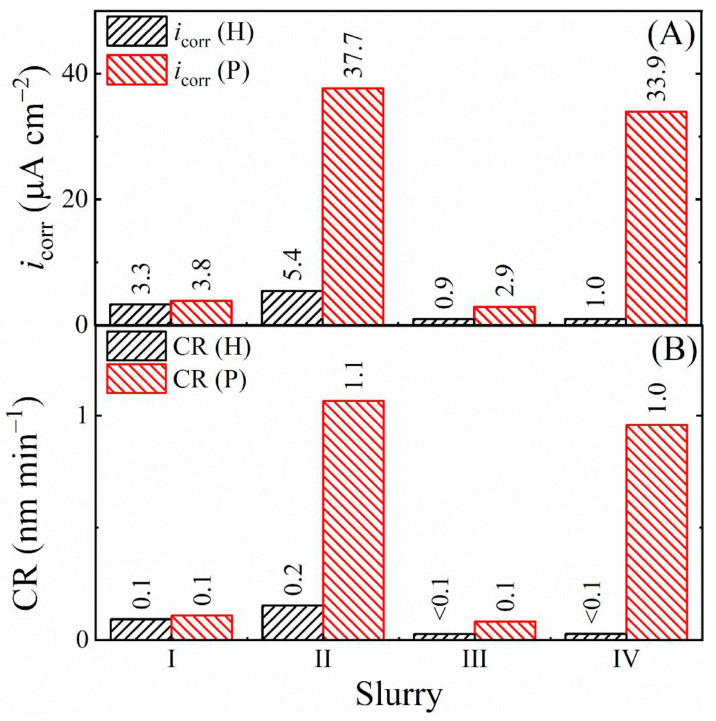
(**A**) comparison of corrosion current densities (*i*_corr_) and (**B**) corrosion rates (CR) of each test slurry under hold (H) and polish (P) conditions with 0.0142 MPa down pressure on the sample surface applied against the polishing pad. The values of CR (H) in slurries III and IV (0.026 and 0.027 nm min^−1^, respectively) have been rounded off to maintain consistency of significant figures in the figure. The mutually comparable values of CR (P) observed between slurries II (without H_2_O_2_) and IV (with H_2_O) indicate how mechanical abrasion adds a strong component of wear induced corrosion. The CMP supporting surface film of SS does not effectively form in sully I, and for this reason, the function of abrasion in surface film removal is relatively subdued for this slurry.

The relative effects of (electro)chemical reactions and mechanical abrasion on the rate of corrosion-like surface modification of SS are examined in Figure 9. Panel (A) shows the corrosion current densities determined from the PDP plots in Figure 7 for the SS test sample in the Down-Hold (H) and Down-Polish (P) arrangements. *i*_corr_ (H) and *i*_corr_ (P) denote the corrosion current densities measured for the Down-Hold and Down-Polish sample configurations, respectively.

The corrosion rates (CRs) corresponding to the *i*_corr_ data in Figure 9A were determined from the following formula which is commonly used for alloys [111]:

(26) $$CR=\frac{i_{corr}}{\rho F}\times \frac{1}{\sum_{i}\;\left(\frac{z_{i}}{w_{i}f_{i}}\right)}$$

where *ρ* and *F* are the density of SS316 and the Faraday constant, respectively. *z*_i_, *w*_i_, and *f*_i_ represent electron valence, atomic mass, and mass fraction of the *i*th element. Only the elements contributing >1 wt% of the SS sample (Fe, Cr, Ni and Mo) were included here in the calculation of CRs, and the results are shown in Figure 9B. The CRs for hold [CR (H)] and polish [CR (P)] were determined by using the *i*_corr_ (H) and *i*_corr_ (P) data in Equation (26), respectively.

The values of CR (P) are larger than those of CR (H) for all the four slurries, since polishing the SS sample reduces the level of surface passivation by removing the surface film (and the Fe-oxide, which mostly forms in slurries I and II). For the reason of abrasive-supported efficient polishing, the MRRs in the alumina containing slurries II and IV are higher than their counterparts found in the abrasive free slurries I and III. In slurry I, CR (H) and CR (P) appear at mutually comparable low values and show that polishing has no measurable effects on the corrosion rates in this case.

**Figure 10:**
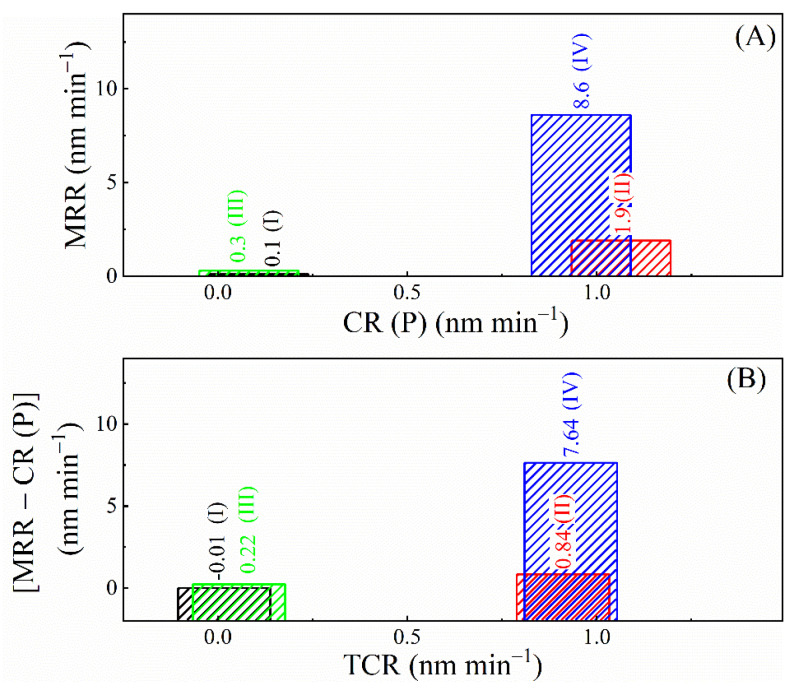
Examination of corrosion and tribo-corrosion as underlying processes of CMP-specific material removal. (**A**) Correlation between material removal rates and corrosion rates measured during surface polishing in four test slurries (I)–(IV). (**B**) Difference between the rates of material removal and polish-supported corrosion correlated with the tribo-corrosion rate for a SS CMP sample in slurries (I)–(IV). The numbers associated with the bars denote the corresponding plotted values.

To further probe the tribo-electrochemical mechanism of CMP, it is necessary to check how the values of MRRs compare to those of CR (P). This is achieved in Figure 10A where the MRRs taken from Figure 1 are plotted against CR (P) values taken from Figure 9B. The oxidizer-dependent variations in the MRRs strongly correlate with those of the CR(P)s both in the presence and in the absence of alumina abrasives. Moreover, in all four slurries, the MRRs are notably higher than their corresponding CR(P)s. Similar differences between the rates of material removal and surface corrosion have been frequently observed in previous studies of metal CMP [38,60,112]. Such gaps between MRRs and CR(P)s [i.e., *MRR − CR(P) >* 0] can be explained by accounting for the last two terms in Equation (3).

In metal CMP, the process of tribo-corrosion (value of *R*_wc_) often controls the value of *R*_cw_ in a manner such that *R*_cw_ linearly varies with variations in the TCRs. If *R*_cw_ >> *R*_w_ (which is a commonly observed situation [50]) in such cases, the values (of MRR and those) of [*MRR* − *CR*(*P*)] exhibit a correlation of proportionality with slurry-dependent values of TCRs in such cases [38]. This effect for the present CMP system is checked in Figure 10B, where the slurry-dependent values of [*MRR* − *CR*(*P*)] from Figure 10A are plotted against those of their corresponding TCRs obtained from the CR data in Figure 9B. According to this plot, the rate differences [*MRR* − *CR*(*P*)] are indeed correlated to the TCRs, suggesting that the contribution of *R*_cw_ likely dictates the difference between the values of MRRs and CR(P)s. The relative magnitudes of the MRRs and TCRs also indicate that the chemically modified material itself has a relatively small contribution to the MRRs measured in CMP, while surface regions (likely adjacent to and) affected by the chemically reacted surface sites supply most of the material removed by abrasion.

### 3.9. Collective Implications of the Results of Tribo-Electrochemical Measurements

The results of the muti-technique measurements presented in Section 3.4, Section 3.5, Section 3.6, Section 3.7 and Section 3.8 provide a detailed account of the CMP enabling surface processes as detected by the different electrochemical methods used. A complete description of the operative mechanisms of material removal can be assembled by briefly summarizing the main findings and the analytical steps leading to these conclusions; this description is presented below.

According to Figure 1, the combined actions of the complexing agent CB and the oxidizer H_2_O_2_ are necessary to chemically prepare the SS surface for material removal. These data also indicate that an active mechanical component of surface abrasion by alumina particles is necessary to effectively remove material from the (electro)chemically modified SS surface. The surface reactions governing the chemical component of CMP are considered in Equations (6)–(23). Reactions (6)–(9) represent metal dissolutions in the acidic slurry according to Pourbaix diagrams of the affected metals. Due to the absence of measurable ER values, it is evident that the metal cations generated in these reactions form insoluble surface complex films (with the identity of salt films), which represent part of the material removed here in CMP.

EIS data collected in four compositionally varied slurries exhibit impedance features that are consistent with those of typical salt films formed on metal/alloy surfaces in acidic solutions. Comparative results of LPR experiments performed with and without surface polishing show once again that abrasive particles are necessary to effectively remove surface layers of the SS sample. This active role of alumina abrasives in material removal is further verified by intermittent OCP transients recorded in alternated cycles of polishing and static holding. OCP measurements performed using a separate sample configuration of Up-Rotation show that the hydrodynamic effects alone of a moving slurry in the CMP setup do not contribute to the process of material removal from the SS sample.

The rate-correlation plots shown in Figure 10 suggest that the process of salt film formation on the SS surface has a key function in activating corrosion-induced wear. Due to the wear mechanism, most of the material removed by abrasion in this CMP study likely comes from surface regions that are structurally affected by but not included within the salt film. In comparison, the film itself represents a relatively small fraction of the removed material. This observation is notable in terms of expanding the current understanding of SS CMP mechanisms, since surface modification using oxidizers and complexing agents is often considered as a primary mode of material removal in metal/alloy CMP. Other cases with such minor direct contributions of electrochemical corrosion to material removal have been previously reported by Choi et al., Stein et al., and Cui et al. in their CMP studies of Cu [60], W [112], and Ru [113] systems, respectively. CMP mechanisms of this type tend to create a notable gap between the values of corrosion rates and MRRs measured under polishing. This gap is observed here in Figure 10 and analyzed in terms of distributed modes of material removal using the description of Equation (3).

## 4. Conclusions

CMP of SS is of considerable current interest for its broad-ranging applications in modern metallographic processing of stainless steel. However, optimization of the slurry chemistries for SS CMP is associated with several challenges, as the tribo-electrochemical mechanisms of material removal for these systems have remained relatively underexplored in the literature. The experiments reported in this work focus on unveiling these mechanisms of SS CMP using a model system based on 316/316L SS samples and acidic polishing slurries. The main test slurry here contains a low concentration (1 wt.%) of H_2_O_2_ as an oxidizer, with alumina abrasives and CB functioning simultaneously as a pH adjuster, a complexing agent, and a dispersant for the abrasive particles. Based on its chemical composition, the overall environmental compatibility of this slurry is comparable to those previously used in studies focused on sustainable methods of CMP [114].

A specific challenge of slurry formulation for SS CMP is that these systems typically require acidic slurries, but the constituent metals of the alloy tend to dissolve in acidic media. The presence of dissolution in CMP is generally manifested in the form of non-negligible etch rates, and minimizing this dissolution to avoid surface defects often becomes a required feature of the polishing slurry [15,18,31]. The results presented here show that this issue of uncontrolled dissolution can be effectively addressed by converting the dissolution-prone surface layers to mechanically removable porous salt films with suppressed solubility. The presence of CB in the CMP slurry of this study enables the formation of these salt films. When added to the slurry, H_2_O_2_ further augments the creation of these surface films, and alumina abrasives promote the films’ removal through abrasion.

The reaction mechanisms considered for surface complex formation by CB are supported by EIS results. According to the film-forming reactions [Equations (20)–(23)], all the main metal components of SS 316/316L contribute to the salt film’s composition, allowing relatively non-selective formation of the latter at the alloy surface. This is a preferred feature of complexing agents for alloy CMP to support spatially homogeneous material removal. The surface complexing role of CB is also manifested as that of a dissolution suppressor, and this helps to further improve the environmental aspect of slurry formulation by allowing the exclusion of separate corrosion inhibitors like BTA from the slurry.

Multiple techniques of tribo-electrochemistry are employed here under closely mimicked experimental conditions of fab-based CMP to investigate the different modes and mechanisms of material removal. The main findings of these experiments are summarized in Section 3.9, which outlines the predominant mechanistic features of material removal. According to these results, the process of surface complex formation is responsible for activating two parallel modes of material removal through corrosion-like material wear and induced wear in uncorroded surface regions, where the latter mode is comparatively more prevalent. To explore if the CMP mechanism analyzed in this study also applies to other systems of SS CMP, it will be necessary to study different grades and families of SS samples in an expanded framework of the analytical approach discussed here.

In view of the above results, the implications of the present experimental approach can be noted in a broader context of studying CMP mechanisms for various alloys other than SS. Based on the current applications of CMP in alloy processing, CMP systems involving TaW [115], Ti_6_AlV [116,117] and various Al-based alloys [118,119,120] could be potential candidates for such investigations to facilitate slurry-engineering for these systems. Like most other reported studies of laboratory scale CMP systems [5,6,9,12,13,18], the experimental setup reported here is limited to examinations of relatively small (2.54 cm diameter disk) CMP samples. Therefore, scaling up these measurements to larger samples for potential industrial applications will require modifications of the current experimental design [26]. It should be possible, however, to implement most of the protocols illustrated here for analyzing CMP-specific tribo-electrochemical data in the general framework of data processing for such scaled-up experimental systems.

## Figures and Tables

**Table 1:** Elemental composition of SS316/316L by median percentages of constituent elements.

Element	Fe	Cr	Ni	Mo	Mn	Si	Cu	Ti	S	N	C	P
Median %	65.9	17.5	12.5	1.50	1.00	0.50	0.50	0.35	0.18	0.05	0.04	0.02

**Table 2:** Naming conventions, compositions, and solution resistances of experimental CMP slurries.

Slurry	Composition	*R*_S_ (Ω)
I	0.1 M KNO_3_ + 0.1 M CB (Ref)	59.1
II	Ref + 3 wt% 0.3 μm Alumina	65.9
III	Ref + 1 wt% H_2_O_2_	75.8
IV	Ref + 1 wt% H_2_O_2_ + 3 wt% 0.3 μm Alumina	66.6

**Table 3:** Designations of Test Systems Based on Sample Setup Configurations.

Configuration of Sample Setup	System Designation
(a) SS sample surface lifted by 1 mm above the polishing pad maintaining sample surface parallel to the pad surface. Both the sample and the pad are held stationary.	Up-Hold
(b) SS sample surface lifted by 1 mm above the polishing pad maintaining sample surface parallel to the pad surface. Both the sample and the pad are rotated at a common angular speed of 95 rpm.	Up-Rotate
(c) SS sample surface pressed down onto the polishing pad at a pressure of 0.014 MPa, while both the sample and the pad are held stationary.	Down-Hold
(d) SS sample surface pressed down onto the polishing pad at a pressure of 0.014 MPa, while both the sample and the pad are rotated at a common angular speed of 95 rpm.	Down-Polish

**Table 4:** Impedance parameters for the SS CMP surface obtained from CNLS analyses of EIS data recorded in the Up-Hold and Down-Hold sample configurations.

EEC Parameter *	CMP Systems (Up, Down)			
	I	II	III	IV
*R*_s_ (Ω), *R*_sc_ (Ω cm^2^)	59.1, 1704	65.9, 1254	75.8, 1210	66.6, 1091
*C*^o^_Fc_ (μF cm^−2^)	82.1	79.1	1.9	2.3
*R*^o^_Fc_ (kΩ cm^2^)	60.7	19.1	0.21	0.17
τ^o^_Fc_ (ms)	4983	1511	0.40	0.39
*C*_F_, *C*_Fc_ (μF cm^−2^)	8.94, 0.80	5.28, 0.81	3.74, 5.41	5.19, 5.87
*R*_F_, *R*_Fc_ (Ω cm^2^)	20.5, 87.1	21.7, 59.1	42.1, 1152	68.4, 1097
*Y*_d_, *Y*_dc_ (μS s^d^ cm^−2^)	24.0, 115.4	15.2, 139.5	6.2, 21.2	6.4, 21.8
*d*, *d_c_*	1, 0.54	1, 0.54	1, 0.85	1, 0.85
*R*_a_ (Ω cm^2^)	66.5	63.9	87.0	88.9
*Y*_a_ (μF cm^−2^)	83.3	48.3	14.1	13.4
*C*_a_ (μF cm^−2^)	26.63	16.05	3.93	2.01
*a*	0.82	0.84	0.82	0.78
*τ*_a_ (ms)	1.77	1.02	0.34	0.18
*R*_p_, *R*_pc_ (kΩ cm^2^)	62.0, 2.71	146, 2.00	81.2, 147	79.2, 144

* The impedance elements representing these parameters are displayed in the EECs in Figure 2A,B (insets). The rows containing two entries (separated by a comma) in each cell of this table represent the values of the corresponding variable measured in the Up-Hold (first entry) and Down-Hold (second entry, labeled with a subscript “c”) situations, respectively.

## Data Availability

The original contributions presented in this study are included in the article/Supplementary Material. Further inquiries can be directed to the corresponding author.

## Supplementary Materials

The following supporting information can be downloaded at: https://www.mdpi.com/article/10.3390/ma18020317/s1. Figure S1: Nyquist impedance plots for the SS CMP sample, recorded in test slurries I, II, III and IV, in the Down-Hold and Up-Hold configuration. The figure panels are labeled according to their corresponding slurry designations. The data-stabilities observed here indicate structural stabilities of surface films formed on the SS sample under stationary hold; Figure S2: Steps of processing PDP (Tafel) data that were recorded in the Down-Polish sample configuration and contained current fluctuations (noise) of tribo-corrosion; Table S1: Statistical Errors in Impedance parameters Obtained.
