# Supplementary material for: Tribo-Electrochemical Mechanism of Material Removal Examined for Chemical Mechanical Planarization of Stainless-Steel Using Citrate Buffer as a Complexing Agent
Source: Materials (Basel). 2025 Jan 12;18(2):317. doi: 10.3390/ma18020317 (PMC11767002; doi:10.3390/ma18020317)
Supplement: Supplementary file 1 [file materials-18-00317-s001.zip › materials-3396790-supplementary.pdf]

Supplementary Materials

Tribo-Electrochemical Mechanism of Material Removal Examined for Chemical Mechanical Planarization of Stainless-Steel using Citrate Buffer as a Complexing Agent

D. R. Santefort. K.U. Gamagedara and D. Roy*

Department of Physics, Clarkson University, Potsdam, NY 13699-5820, U.S.A.
*Correspondence: droy@clarkson.edu

S.1. Stability and Repeatability Characteristics of Impedance Data

Temporal stability of is a required feature of valid EIS data [1]. If the frequency range of EIS perturbation is restricted by system requirements, EIS stability can be checked experimentally by preforming repeated measurements of impedance spectra without changing the experimental conditions of the interface, and then by checking for variations among the spectra collected at different times. CMP interfaces involving arrangements for tribo-electrochemical measurements tend to be in the category. The high-frequency limit of an impedance spectrum in such cases is limited to prevent inductive effects of the experimental set up, while the corresponding low-frequency limit is restricted for avoiding surface modifications due to prolonged data collection times that substantially exceed the usual polishing times.

EIS stability tests based on comparison of repeatedly recorded spectra were performed for the SS CMP systems used in this study. Each experimental sample-slurry contact was established by allowing the system's initial OCP to arrive at a steady state, which typically required ~5 min. Three repeated cycles of EIS spectra of the CMP interface were collected at the steady state OCP. The obtained results of these EIS tests for the up-Hold and Down-Hold sample settings in the four test slurries are displayed in Figure S1.

Each panel in Figure S1 corresponds to an individual slurry, and three consecutively collected Nyquist spectra are shown for two specific sample-configurations. The consecutive Nyquist plots, labeled as cycles 1, 2 and 3 (according to the sequence of their recording) are layered in the figure following the order of their cycle numbers. As seen in Figure S1, the data for all three cycles in each sample configuration strongly overlay on each other; thus, the plot for cycle 3 being placed in the final layer largely obscures the plots of the of cycles 1 and 2. This strong overlap of the three cycles of EIS data confirms spatial stability of the data. CNLS analyses of the Nyquist plots from all three cycles resulted in identical EEC models, and indicated signature features of stable salt films as discussed in the context of Figure 2.

The temporal stability of EIS observed in Figure S1 also implies that the removable surface films formed on the SS CMP sample remain structurally stable under stationary hold conditions. For the frequency spectrum used in this work, the EIS recording time for each cycle was about 3 min. As shown in Figure 3, the total times for the surface films to form and stabilize under hold conditions are substantially shorter than this timescale of data scan. Since the Nyquist spectra do

not change with repeated data-scans, it is evident that the surface film in each stationary case remains structurally stable upon its formation. This observation is consistent with the ~zero etch rates found in dissolution measurements. The main framework of data analysis

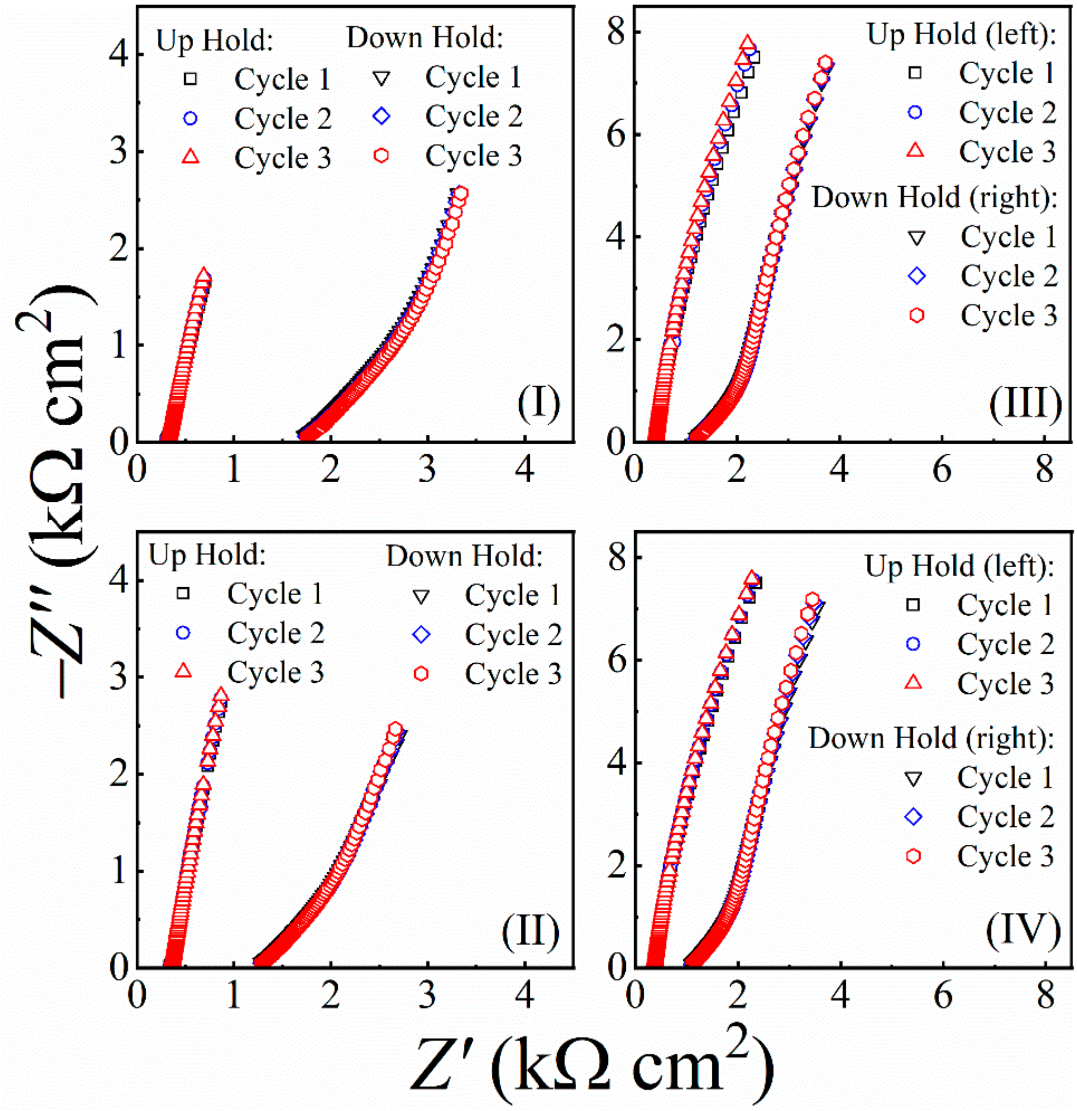

Figure S1. Nyquist impedance plots for the SS CMP sample, recorded in test slurries I, II, III and IV, in the Down-Hold and Up-Hold configuration. The figure panels are labeled according to their corresponding slurry designations. The data-stabilities observed here indicate structural stabilities of surface films formed on the SS sample under stationary hold.

in this study is based on treating the salt film of SS as an insoluble, removable material. The above findings based on the EIS data in Figure S.1 further solidify this point.

S.2. Statistical Errors in Electrochemical Impedance Parameters

The CNLS analyzed impedance variables obtained from the EIS data in Figure 2 are presented in Table 4. The statistical uncertainties in each of the fitted elements are presented here in Table S1 using the notations introduced in the discussion of Figure 2 in the main text. The raw data (taken from three repeated sets of measurements) for this analysis are shown in Figure S1. The percentile errors listed in Table S1 are obtained as:

$$\% \, Error = \frac{\text{Standard error}}{\text{Estimated best (mean)value}} \times 100$$

The ZSimpWin Software was used to calculate these errors in the complex nonlinear least square (CNLS) approach, and the mathematical protocols used for these calculations are described in detail in an earlier report by Boukamp [2].

Table S1. Statistical Errors in Impedance parameters Obtained.

Impedance Parameter (Unit)	Percentile Errors in Impedance Parameters (%) CMP Systems (Up, Down)			
	I	II	III	IV
R_s (Ω), R_{sc} (Ω cm^2)	0.15, 0.12	0.24, 0.16	0.11, 0.26	0.13, 0.25
C_{Fc}^0 (μF cm^{-2})	0.72	0.50	3.83	4.10
R^0_{Fc} (Ω cm^2)	10.8	1.99	2.82	3.00
C_F, C_{Fc} (μF cm^{-2})	6.84, 5.19	11.4, 7.17	3.78, 2.20	2.49, 2.10
R_F, R_{Fc} (Ω cm^2)	2.99, 2.95	3.43, 3.43	4.71, 2.51	2.81, 2.73
Y_d, Y_{dc} (μS s^d cm^{-2})	0.73, 1.40	0.94, 1.31	2.17, 2.03	12.0, 2.02
d, d_c	0.20, 0.61	0.27, 0.58	1.10, 0.69	1.92, 0.69
R_a, (Ω cm^2)	2.27	2.05	17.0	49.3
Y_a (μF cm^{-2})	1.47	1.68	5.54	8.31
a	0.30	1.30	0.54	0.23
R_p, R_{pc} (Ω cm^2)	3.86, 2.28	3.63, 1.99	2.11, 19.0	3.57, 18.9

* The rows containing two entries (separated by a comma) in each cell of this table represent the values of the corresponding variable measured in the Up Hold (first entry) and Down Hold (second entry, labeled with a subscript "c") situations, respectively.

As seen from the results presented in Table S1, the errors found in most cases are limited below 10%. Two exceptions from this general trend can be noted for the uncertainties of R_a in slurries III and IV. As seen in the Nyquist plots in Figure S1, the overall impedance (especially the

imaginary component of impedance nearly doubles) and extends further into the low-frequency spectral in going from slurries I and II. As explained in the discussion of Figure 2, the impedance of the CMP interface increases in slurries II and IV as the H_2O_2 included in these slurries augment the formation of passive salt films on the SS sample surface. Nevertheless, due to practical requirements (specified in [3]) associated with the three electrode CMP system tested, the usable spectral range of EIS remains limited for all four slurries. At the same time, since an extended frequency range is necessary to adequately analyze experimental EIS spectra in the CNLS approach, fitting errors resulting from these calculations are expected to increase while operating in a restricted frequency range [2]. In the present case, this restriction mostly affects the low frequency EIS spectra for slurries II and IV, and the resulting statistical uncertainties are manifested here in the values of the adsorption resistance.

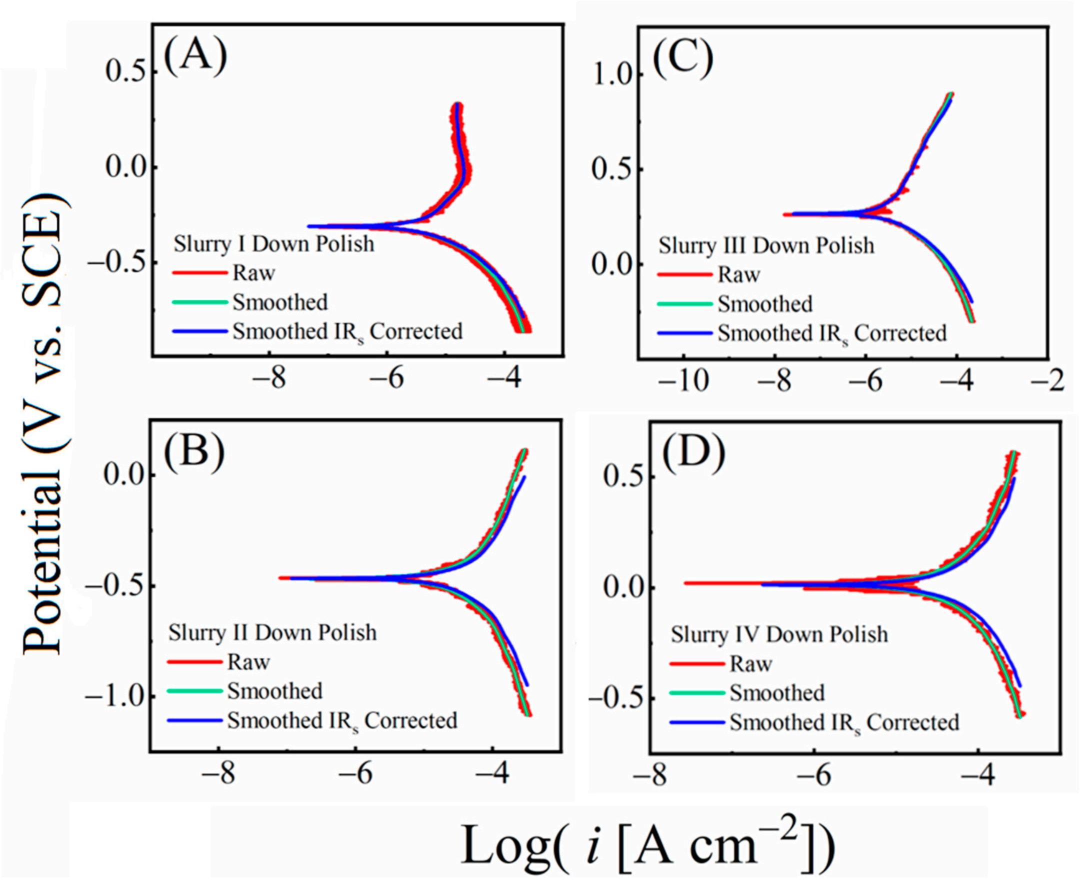

Figure S2. Steps of processing PDP (Tafel) data that were recorded in the Down-Polish sample configuration and contained current fluctuations (noise) of tribo-corrosion.

S.3. Processing of Potentiodynamic Polarization Plots Recorded in the Down-Polish Sample Configuration

Figure 6 in the main report showed signature noise features (potential fluctuations) of tribo-corrosion in the measurements of OCP transients using the Down-Polish sample set up. Characteristic tribo-noise of this set up was also detected as current fluctuations in the PDP data

that were superimposed on the polarization plots. While the noise-levels were moderate, it was necessary to smoothen the data for the Tafel Extrapolation software to function adequately. The detailed protocol of data processing in this approach has been described previously [4]. The main steps of this analysis for the systems studied in this work are briefly elaborated here using the data shown in Figure S2.

The raw data containing tribo-noise (shown in red) was smoothened using the LOWESS procedure in Origin. The smoothened data (shown in green) were corrected for the ohmic potential drop IR_s. These smoothened and corrected plots were used for Tafel extrapolation to determine the corrosion variables.

References

1. Turk, M.C.; Walters, M.J.; Roy, D. Experimental considerations for using electrochemical impedance spectroscopy to study chemical mechanical planarization systems. *Electrochimica Acta* **2017**, *224*, 355-368.
2. Boukamp, B.A. A Nonlinear Least Squares Fit procedure for analysis of immittance data of electrochemical systems. *Solid State Ionics* **1986**, *20*, 31-44.
3. Gamagedara, K.; Roy, D. Mechanisms of Chemically Promoted Material Removal Examined for Molybdenum and Copper CMP in Weakly Alkaline Citrate-Based Slurries. *Materials* **2024**, *17*, 4905.
4. Gamagedara, K.; Roy, D. Experimental Strategies for Studying Tribo-Electrochemical Aspects of Chemical–Mechanical Planarization. *Lubricants* **2024**, *12*, 63.
